# *CETP* gene polymorphisms and haplotypes are explanatory variables for HDL cholesterol level in sickle cell disease

**DOI:** 10.1590/1414-431X2023e12879

**Published:** 2024-01-22

**Authors:** N.R.C. Cruz, T.N.S. Valente, F.O. Ferreira, L.R. Macedo, A.R. Belisário, C.M. da Silva, N.S. Oliveira, A.F.F. Gomides, C. Velloso-Rodrigues

**Affiliations:** 1 Laboratório de Biologia Celular e Genética Molecular, Departamento de Nutrição, Universidade Federal de Juiz de Fora - Campus Governador Valadares, Governador Valadares, MG, Brasil; 2 Laboratório de Biologia Celular e Genética Molecular, Departamento de Ciências Básicas da Vida, Universidade Federal de Juiz de Fora - Campus Governador Valadares, Governador Valadares, MG, Brasil; 3 Departamento de Ciências Básicas da Vida, Universidade Federal de Juiz de Fora - Campus Governador Valadares, Governador Valadares, MG, Brasil; 4 Departamento de Economia, Universidade Federal de Juiz de Fora - Campus Governador Valadares, Governador Valadares, MG, Brasil; 5 Centro de Tecidos Biológicos, Fundação Hemominas, Lagoa Santa, MG, Brasil; 6 Faculdade de Ciências Médicas de Minas Gerais, Belo Horizonte, MG, Brasil

**Keywords:** Sickle cell disease, *CETP* gene, Alpha-thalassemia, Lipoproteins, Apolipoprotein

## Abstract

Variations in lipid profile have been observed in sickle cell disease (SCD) and understanding their relationship with disease severity is crucial. This study aimed to investigate the association of polymorphisms of the *CETP* gene and laboratory markers of disease severity with lipid profile in a pediatric population with SCD. Biochemical and anthropometric analyses and *CETP* and alpha-thalassemia genotyping were performed. The study included 133 children and adolescents with sickle cell anemia (SCA) or hemoglobin SC disease (SCC), in steady-state. The SCA and no hydroxyurea (no HU) groups had higher values of ApoB, total cholesterol, low-density lipoprotein cholesterol (LDL-C), and non-high-density lipoprotein cholesterol (non-HDL-C) compared to the SCC and HU groups. However, there were no significant differences in ApoA1 and HDL-C levels between the groups based on genotype. Furthermore, the groups with altered levels of ApoA1, HDL-C, and the triglyceride/HDL ratio exhibited lower hemoglobin (Hb) levels and higher white blood cell counts. Hb level was associated to HDL-C levels. Analysis of *CETP* gene variants showed that the minor alleles of rs3764261 (C>A), rs247616 (C>T), and rs183130 (C>T), as well as the TTA haplotype, are explanatory variables for HDL-C levels. These findings suggested that dyslipidemia in SCD, specifically related to HDL-C levels, may be influenced by individual genetic background. Additionally, further investigation is needed to determine if clinical manifestations are impacted by *CETP* gene variants.

## Introduction

Sickle cell disease (SCD) is a common, severe form of inherited hemoglobinopathy with widely distributed global prevalence and high death rates in Brazil (1). It is characterized by chronic hemolytic anemia, inflammatory response, and oxidative stress within the microcirculation (2). Laboratory markers of SCD severity include plasma hemoglobin (Hb), platelets, lactate dehydrogenase (LDH), bilirubin, reticulocytes, and white blood cells (3). In addition, changes in lipid profile are observed, with reductions in serum levels of high-density lipoprotein cholesterol (HDL-C) and an increase in triglycerides (TG), characterizing dyslipidemic syndrome (4,5).

Recent advances have been observed in the treatment of SCD, however, some controversies regarding modifying factors of the risk of pathophysiological vascular changes remain.

The observation of the lowest Hb levels and the highest nitric oxide metabolites in individuals with sickle cell anemia (SCA) with low HDL-C levels (HDL-C <40 mg/dL) (6) suggests that hypocholesterolemia may be related to pathophysiological factors of the disease itself, such as high erythropoietic activity because of the increased demand for cholesterol (7). Hemolysis, oxidative stress, and inflammation in SCD cause reduction in HDL-C and Apo A1 levels, and it has been suggested that this is due to dysfunction and slowed functioning of the reverse cholesterol transport (4). Another explanation is that oxidative stress in SCD promotes the transformation of HDL-C into pro-inflammatory HDL-C (8).

On the other hand, heritability in determining the lipid profile in individuals without SCD was estimated to be between 27 to 48% for HDL-C (9), with the *CETP* gene being a strong candidate in the study of variations in this lipid trace (10). This gene, located on chromosome 16q13 (MIM 118470), encodes the cholesterol ester transfer protein (CETP), which facilitates the transfer of cholesteryl esters from HDL-C to particles containing apolipoprotein B (ApoB), such as low-density lipoprotein cholesterol (LDL-C), in exchange for TG (11). The increase in CETP activity has been associated with a reduction in HDL-C levels. Alleles of the *CETP* gene are involved in the increased expression of CETP (11) or HDL-C levels (12-14). A significant number of common variants of the *CETP* gene have effects on the level or activity of CETP, among them are rs3764261 C/T, rs247616 C/T (11,15), and rs183130 C/A (16).

Understanding the mechanisms and the modifying factors involved in dyslipidemia in SCD may impact the prognosis of complications associated with morbidity and mortality and help define therapeutic targets. Although some studies identify alterations in the lipid profile in children with SCD (3,5), little research has been done on the risk factors. Furthermore, no research has been found on the relationship of dyslipidemia with *CETP* gene polymorphisms in this group. In this work, we investigated the associations of *CETP* gene polymorphisms and laboratory markers of severity with lipid profile to contribute to a better understanding of the pathophysiological aspects resulting from lipid homeostasis in SCD.

## Material and Methods

### Study design and populations

This was an observational descriptive cross-sectional study carried out between September 2015 and July 2019. It was conducted in an outpatient clinic of the Hemominas Foundation in Minas Gerais (Brazil). The study included all children and adolescents homozygous for HbS (sickle cell anemia; SCA, HbSS) or heterozygous for the S and C alleles (hemoglobin SC disease, SCC), in steady-state, who were taking hydroxyurea (HU) and those who were not. Exclusion criteria were use of drugs known to affect lipid metabolism, presence of other chronic illnesses not related to SCA, blood transfusion in the three months that preceded blood collection, and gestation.

### Ethics statement

Participants were informed about study objectives and of their right to refuse to take part in the study or withdraw from the study without consequences for their follow-up at the Hemominas Foundation. Written informed consent from parents and participants were obtained before enrollment. The study followed the guidelines of the Declaration of Helsinki (1964). It was approved by the Ethics Committees of the Federal University of Juiz de Fora (number 2.146.315) and Hemominas Foundation (approval numbers 1.137.657 and 2.521.811).

### Anthropometric evaluation

Weight and height were measured using an electronic scale (Líder^®^, Brazil) and an anthropometer (Altura Exata^®^, Brazil) with the child wearing light clothes and standing barefoot. Body mass index (BMI) was calculated as weight (kg)/height^2^ (m^2^). Weight and height were transformed into z-scores for BMI/age and sex (BMI z-score) and classified using WHO AnthroPlus software (WHO media products, Switzerland) (17). Children whose BMI z-score was <−2 standard deviations (SD) below the mean were classified as underweight, ≥−2 to ≤1 SD as normal weight, and >1 SD as overweight or obese.

### Biochemical and hematological measurements

After an overnight fast (8-12 h), venous blood was drawn into tubes with gel separator. Blood samples for biochemical and hematological analyses were obtained on the same day through a single forearm puncture on the day of the routine doctor appointment. Samples were immediately analyzed. Extraction of hematological results occurred after they were recorded in the medical records. TG, total cholesterol, and HDL-C were measured using enzymatic kits (Labtest^®^, Brazil) according to the manufacturer's instructions. Non-high-density lipoprotein (non-HDL-C) was obtained by the difference between total cholesterol and HDL-C concentrations. Apolipoprotein A1 (ApoA1) and ApoB were measured by immunoturbidimetric assays accordingly (Abbott^®^, Brazil). Total bilirubin and direct bilirubin were directly measured by the Sims/Horn method using enzymatic kits (Labtest^®^), and indirect bilirubin was calculated by the difference between total bilirubin and direct bilirubin. LDH was measured by the enzymatic colorimetric method, according to the manufacturer's instructions. All biochemical measurements were performed automatically using the Cobas Mira Plus equipment (Roche, Brazil). Hb, white blood cells, and platelets were measured on the ABB Micros 60, HORIBA^®^ (Brazil). Hb, LDH, bilirubin concentrations, and white blood cells count were used as SCD laboratory markers of severity.

### Genotyping of the *CETP* gene and alpha-thalassemia

Genomic DNA (gDNA) was extracted from the buffy coat of peripheral blood using a QIAamp DNA Blood Mini Kit (QIAGEN^®^, Germany) according to the manufacturer's recommendations. gDNA was quantified spectrophotometrically using NanoVue Plus GE^®^ (Germany), and the concentration of gDNA was normalized to 5 ng/µL.

Three single nucleotide polymorphisms (SNPs) of the *CETP* gene were selected for the study: rs3764261 (CC *vs* CA+AA), rs183130, and rs247616 (CC *vs* CT+TT). This choice was based on the frequency of rare alleles in the population and the high linkage disequilibrium between them. Previous publications on the effects of these SNPs on CETP activity and HDL-C levels in individuals without sickle cell disease (11,12,15) as well as the lack of studies with the sickle cell disease population were also considered.

Genotyping of SNPs of the *CETP* gene was performed by fluorescence-based allelic discrimination using TaqMan^®^ 7500 Real-Time PCR platform (Applied Biosystems^®^, USA). The reaction mix was composed by ultrapure water (0.5 μL per sample), TaqMan^®^ SNP Genotyping Assays (20X, 0.5 μL per sample), TaqMan™ Genotyping Master Mix (2X, 5 μL per sample), and template DNA (20 ng). The results of the amplification and determination of genotypes were obtained using the Thermo Fisher Cloud platform (available at https://apps.thermofisher.com/apps/dashboard/#/).

In addition, −α^3.7^-thalassemia and −α^4.2^-thalassemia detection were investigated in the *HBA1* and *HBA2* genes by multiple gap-PCR following a previously published procedure (18).

### Statistical analysis

Categorical variables are reported as frequencies and percentages and were compared using the chi-squared test. Shapiro-Wilk statistic was used to test the normality of the distribution of metric data. The normally distributed data are reported as means±SD and non-normally distributed data are reported as the median and first/third quartiles. Two-tailed independent samples *t*-test, Mann Whitney test, and Spearman/Partial correlation test were used.

Mean values of laboratory markers of severity were compared between groups according to genotype (SCA *vs* SCC), drug therapy (no HU *vs* HU), and lipid fractions (adequate levels *vs* altered). For the categorization of lipid levels according to the pathologically altered levels (low or high), the reference values proposed by the Expert Panel on Integrated Guidelines for Cardiovascular Health and Risk Reduction in Children and Adolescents were considered (19). This reference considers two age groups, below and above 10 years of age, for TG and classifies lipid markers into adequate (acceptable and borderline) and altered (low or high). The TG/HDL-C ratio was calculated and classified according to Barbalho et al. (20).

Subsequently, the association between the investigated *CETP* polymorphisms and lipid fractions were analyzed.

Hardy-Weinberg equilibrium was determined by the application available at https://wpcalc.com/en/equilibrium-hardy-weinberg/.

The dominance effect of the minor allele of each genetic variant was evaluated by grouping the genotypes of rs3764261 (CC *vs* CA+AA), rs183130, and rs247616 (CC *vs* CT+TT). The genotypes were also grouped to analyze the recessive effect (rs3764261: CC+CA *vs* AA; rs183130 and rs247616: CC+CT *vs* TT).

Haplotypes are combinations of specific alleles at different SNPs. The analyses were performed using the Haploview software version 4.2 (https://www.broadinstitute.org) (21), specifically designed for haplotype analysis. This software allows for the visualization and assessment of linkage disequilibrium patterns and haplotype frequencies within a given genomic region. Linkage disequilibrium was estimated using Haploview with the combined data set of all patients. The Haploview program was also used to identify the allele frequency, the association of each SNP variant, and the haplotype blocks with the lipid profile markers in the population. For this analysis, the case groups (altered values - high or low) and control (acceptable and borderline values) of lipid profile markers were considered according to reference values proposed by the National Heart Lung and Blood Institute (19).

Logistic regression was used to verify the association of HDL-C levels with the genotypes of *CETP* SNPs dichotomized into case group (HDL-C <40 mg/dL) and control group (HDL-C ≥40 mg/dL), considering the model dominance. In addition, analyses with haplotype genotypes grouped by presence or absence of the TTA haplotype were performed, including laboratory markers of severity and the HU variable (use or not use). The need to adjust the model for age was verified.

Data were tabulated and analyzed using the Statistical Package for Social Sciences (SPSS) version 20.0 (IBM, USA). The statistical significance level was P<0.05 and the confidence interval was 95%.

### Sample size calculation

Sample size calculation for comparison between groups was performed, considering as reference data obtained by da Guarda et al. (22), comparing Hb data between patients with low HDL and normal HDL. The standard deviation of Hb for patients with low HDL was 0.99. A 0.73 difference in Hb between groups was considered for the calculation. With the calculation, a minimum sample size of 17 participants per group of comparison was obtained, considering 95% statistical significance. In order to allow multivariate analyses and increase the representativeness of the study, the research was developed with a sample size larger than the minimum estimated for comparison between groups. All datasets on which the conclusions were based are available upon request.

## Results

### Characteristics of study participants

This study included 133 participants with SCD, 54.89% (n=73) with SCA and 45.11% (n=60) with SCC. The age range of the patients was 5-17 years with a mean of 11.8±2.8 years. When grouped by age, 52.6% (n=70) of the individuals were between 5 and 10 years old, while adolescents accounted for 47.4% (n=63). Of the total, 55.64% (n=74) were boys and 33.83% were on HU therapy (n=45; 39 with SCA and six with SCC) (Table 1). The duration of HU use in the studied population was over 12 months. Demographic characteristics were similar between the two groups.

**Table 1:** Characteristics of participants stratified by type of sickle cell disease.

Characteristics	All (n=133)	SCA (n=73)	SCC (n=60)	P
Demographic profile				
Age (years)	11.76±2.8	11.57±3.04	12.01±2.54	0.332^*^
<10	52.63 (70)	34.25 (25)	30.00 (18)	0.710^‡^
10-17	47.37 (63)	65.75 (48)	70.00 (42)	
Sex				
Male	55.64 (74)	54.79 (40)	56.67 (34)	0.829^‡^
Female	44.36 (59)	45.20 (33)	43.33 (26)	
HU				
Yes	33.83 (45)	53.42 (39)	10.00 (6)	**< 0.001‡**
No	66.17 (88)	46.58 (34)	90.00 (54)	
Anthropometric profile				
BMI kg/m^2^	16.20 (15.00-18.60)	15.50 (14.85-17.50)	17.25 (15.65-19.25)	**0.003** ^†^
BMI z-score (AU)	-0.52±1.16	-0.78±1.05	-0.19±1.22	0.004*
Underweight	9.77 (13)	10.96 (8)	8.33 (5)	0.223^‡^
Adequate weight	80.45 (107)	83.56 (61)	76.67 (46)	
Overweight	8.27 (11)	5.48 (4)	11.67 (7)	
Obesity	1.50 (2)	-	3.33 (2)	
Alpha-thalassemia^§^				
αα/αα	75.57 (99)	76.39 (55)	74.57 (44)	0.456^‡^
αα/−α^3.7^	22.90 (30)	23.61 (17)	22.03 (13)	
αα/−α^4.2^	0.76 (1)	-	1.70 (1)	
−α^3.7^/−α^3.7^	0.76 (1)	-	1.70 (1)	

SCA: sickle cell anemia; SCC: hemoglobin SC disease; HU: hydroxyurea; BMI: body mass index; AU: arbitrary units. ^§^Two individuals did not participate in the alpha-thalassemia genotyping; n=72 for SCA and n=59 for SCC. ^‡^Results of the chi-squared test are reported as % (absolute number); *Results of the Student's *t*-test are reported as means±SD; ^†^Results of the Mann Whitney's U test are reported as median (first and third quartiles: Q1-Q3). P: comparison between the genotypes. Significant results (P<0.05) are shown in bold.

Children with SCA showed a lower BMI and BMI z-score value. Overall, 9.77% (n=13) were underweight while 8.27% (n=11) and 1.5% (n=2) were classified as overweight or obese, respectively. There was no difference in the classification distribution between the SCA and SCC groups (Table 1).

Alpha-thalassemia was present in 32 individuals, of whom 30 (22.90%) were heterozygous -α^3.7^, one case (0.76%) was -α^4.2^, and one homozygous -α^3.7^/-α^3.7^. There was no difference between the groups (Table 1).

The laboratory parameters stratified according to hemoglobin variant type are shown in Table 2. The SCA group showed higher values of ApoB, total cholesterol, LDL-C, non-HDL-C, TG (in ≥10 years), and TG/HDL-C ratio compared to the SCC group. There was no difference in ApoA1 and HDL-C levels between disease genotypes. As expected, laboratory markers of SCD severity indicated worse clinical status of the disease in the SCA group subjects.

**Table 2:** Laboratory parameters of the participants stratified by type of sickle cell disease.

Parameters	n	SCA (n=73)		n	SCC (n=60)		P
Lipid profile							
ApoA1, mg/dL	73	104.00 (9.50-112.50)		60	110.50 (96.00-122.00)		0.054^†^
ApoB, mg/dL	73	64.64±17.61		60	56.16±12.43		0.002*
TC, mg/dL	73	117.00 (108.00-143.50)		60	113.00 (104.00-126.50)		0.021^†^
LDL-C, mg/dL	73	63.60 (54.10-82.40)		60	60.70 (51.50-69.40)		**0.042** ^†^
Non-HDL-C, mg/dL	73	80.00 (71.00-101.00)		60	74.50 (64.25-84.75)		**0.006** ^†^
HDL-C, mg/dL	73	36.00 (34.00-42.50)		60	38.00 (35.00-45.00)		0.109^†^
TG, mg/dL^§^	73	79.00 (60.50-105.00)		60	71.50 (56.00-89.75)		0.082^†^
<10 years	25	67.00 (54.50-90.50)		18	60.00 (52.00-89.75)		0.571^†^
≥10 years	48	84.00 (66.25-112.25)		42	74.00 (58.75-90.25)		**0.049** ^†^
TG/HDL-C	73	2.16 (1.85-2.81)		60	1.89 (1.50-2.29)		**0.002** ^†^
Laboratory markers of severity							
Hemoglobin, g/dL	69	8.40 (7.50-9.50)		56	11.40 (10.82-12.10)		**<0.001** ^†^
WBC, 10^3^/mm^3^	68	9939.80±4176.85		53	6972.24±2999.17		**<0.001** ^*^
Platelets, ×10^3^/mm^3^	68	478.50 (376.50-543.75)		53	201.00 (164.00-324.50)		**<0.001** ^†^
Total bilirubin, mg/dL	70	2.39 (1.36-3.22)		60	1.17 (0.90-1.96)		**<0.001** ^†^
Direct bilirubin, mg/dL	70	0.53±0.17		60	0.38±0.13		<0.001*
Indirect bilirubin, mg/dL	70	1.77 (0.85-2.56)		60	0.77 (0.59-1.45)		**<0.001** ^†^
LDH, U/L	70	513.00 (425.00-656.75)		60	275.00 (234.00-324.00)		**<0.001** ^†^

SCA: sickle cell anemia; SCC: hemoglobin SC disease; ApoA1: apolipoprotein A1; ApoB: apolipoprotein B; TC: total cholesterol; LDL-C: low-density lipoprotein cholesterol; Non-HDL-C: non-high-density lipoprotein cholesterol; HDL-C: high-density lipoprotein cholesterol; TG: triglycerides; TG/HDL-C: triglyceride/high-density lipoprotein cholesterol ratio; WBC: white blood cells; LDH: lactate dehydrogenase. ^§^The TG variable was analyzed according to age, since the reference value is different for the two age groups. *Results of the Student’s *t*-test are reported as means±SD; ^†^Results of the Mann Whitney’s U-test are reported as median (first and third quartiles: Q1-Q3). Significant results (P<0.05) are shown in bold.

### Relationship between lipid profile and investigated variables

Sex and BMI did not influence the levels of lipid fractions (P>0.05), however, age showed a negative correlation with ApoA1 (r=-0.237; P=0.006), HDL-C (r=-0.290; P=0.001) and a positive correlation with TG (r=0.219; P=0.011) and TG/HDL-C ratio (r=0.185; P=0.033). Thus, the multivariate analysis was adjusted for age.

Association analysis between lipid profile and HU status was performed only with the SCA group, since the number of individuals in the SCC group using this drug was small. Individuals with SCA who used HU presented higher values of ApoA1, ApoB, total cholesterol, LDL-C, and HDL-C. However, HU therapy status did not influence non-HDL-C and TG levels (Table 3).

**Table 3:** Lipid profile of participants with sickle cell anemia stratified by hydroxyurea (HU) treatment status.

Variables	HU (n=39)	No HU (n=34)	P
ApoA1, mg/dL	107.00 (97.00-116.00)	99.50 (94.00-107.00)	**0.041**†
ApoB, mg/dL	68.97±18.04	59.67±15.95	0.023*
TC, mg/dL	124.00 (114.00-148.00)	113.00 (103.75-135.00)	**0.017**†
LDL-C, mg/dL	71.20 (58.80-86.40)	57.10 (49.40-77.45)	**0.016**†
Non-HDL-C, mg/dL	86.00 (73.00-106.00)	74.50 (68.00-101.00)	0.127†
HDL-C, mg/dL	38.00 (35.00-46.00)	35.00 (32.00-41.00)	**0.013**†
TG, mg/dL	70.00 (59.00-102.00)	79.50 (62.25-110.75)	0.394†
TG/HDL-C	2.14 (1.90-2.89)	2.19 (1.80-2.56)	0.965†

ApoA1: apolipoprotein A1; ApoB: apolipoprotein B; TC: total cholesterol; LDL-C: low-density lipoprotein cholesterol; Non-HDL-C: non-high-density lipoprotein cholesterol; HDL-C: high-density lipoprotein cholesterol; TG: triglycerides; TG/HDL-C: triglyceride/high-density lipoprotein cholesterol ratio; HU: hydroxyurea. *Results of the Student's *t*-test are reported as means±SD; †Results of the Mann Whitney's U-test are reported as median (first and third quartiles: Q1-Q3). Significant results (P<0.05) are shown in bold.

Lower TG values were observed in SCD patients with alpha-thalassemia co-inheritance compared to individuals without the deletion (median: 63.00 *vs* 81.00; P=0.041).

ApoB, non-HDL-C, and total cholesterol showed a weak positive correlation with platelets only. ApoA1, HDL-C, TG, and TG/HDL-C ratio showed a weak-to-moderate correlation with at least four laboratory markers of disease severity (Supplementary Table S1). Thus, analyses of the averages/medians of laboratory markers of severity were performed according to the values of these lipid fractions dichotomized into “acceptable/borderline” *vs* “low” for ApoA1 and HDL-C or “high” for TG and TG/HDL-C. Participants with SCD with low ApoA1 levels (<115 mg/dL) had higher levels of laboratory markers of hemolysis (bilirubin, LDH, and Hb) and inflammation (white blood cells). In the SCD group with low HDL-C (<40 mg/dL), Hb levels were lower and white blood cells values were increased. In the SCD group with higher levels of TG (≥100 or 130 mg/dL, according to age), lower levels of Hb and higher platelet count were observed. High levels of bilirubin, LDH, white blood cells, and platelets and low levels of Hb prevailed in the SCD group with high TG/HDL-C atherogenic ratio (>2) (Supplementary Table S2).

### Lipid profile and the *CETP* gene polymorphisms

The analysis of genetic variants of the *CETP* gene showed that the minor allele frequency (MAF) of rs247616 and rs183130 was 0.28, while for rs3764261, it was 0.32.

The genetic variants evaluated were in Hardy-Weinberg equilibrium (P>0.05) and were used to analyze the association with the lipidogram variables. It was observed that the minor alleles of rs247616 and rs183130 were more frequent in the group with the highest level of HDL-C (≥40 mg/dL) (Table 4). There was no significant association with the other lipid fractions.

**Table 4:** Allelic association of variants in the *CETP* gene with HDL-cholesterol levels in participants with sickle cell disease (n=131).

Genetic variant of *CETP*	Minor allele	HDL-C <40 mg/dL/≥40 mg/dL ratio	X^2^	P value
rs247616	T	39 (C):67(T) / 36 (C):120 (T)	5.811	**0.015**
rs183130	T	39 (C):67(T) / 36 (C):120 (T)	5.811	**0.015**
rs3764261	A	41 (C):65 (A) / 45 (C):111 (A)	2.768	0.096

SCD: sickle cell disease; *CETP*: cholesteryl ester transfer protein; HDL-C: high-density lipoprotein cholesterol. Common alleles: rs247616-C, rs183130-C, and rs3764261-C; less frequent alleles: rs247616-T, rs183130-T, and rs3764261-A. Association investigated by the Haploview software (https://www.broadinstitute.org). Significant results (P<0.05) are shown in bold.

The dominance effect of the minor allele of rs3764261 (C>A; genotypes CA+AA *vs* CC) and rs247616 or rs183130 (C>T; genotypes CT+TT *vs* CC) showed an association with higher levels of ApoA1 (P=0.003) and HDL-C (P=0.011) and with lower TG/HDL-C ratio (Table 5). In the recessive model (rs3764261, AA *vs* CA+CC genotypes; rs183130 or rs247616, TT *vs* CT+CC genotypes), there was no relationship with the lipid profile.

**Table 5:** Dominance effects of the SNPs rs3764261, rs183130, and rs247616 of the *CETP* gene in the lipid profile of participants with sickle cell disease.

Variables	Dominant model rs183130 or rs247616 C>T			Dominant model rs3764261 C>A		
	CC (n=58)	CT + TT (n=73)	P value	CC (n=58)	CA + AA (n=73)	P value
	Mean±SDor median (Q1-Q3)	Mean±SDor median (Q1-Q3)		Median (Q1-Q3)	Median (Q1-Q3)	
ApoA1, mg/dL	101.00(93.50-111.50)^†^	108.50(100.00-122.00)^†^	**0.006**	99.00(92.75-110.25)^†^	108.00(100.00-121.50)^†^	**0.003**
ApoB, mg/dL	62.60±17.26*	59.21±14.82*	0.231	59.00(48.00-74.25)^†^	59.00(49.00-67.00)^†^	0.677
TC, mg/dL	115.00(106.50-138.00)^†^	117.00(107.00-135.00)^†^	0.653	113.50(106.75-137.00)^†^	117.00(107.00-135.00)^†^	0.545
LDL-C, mg/dL	61.80(52.90-80.20)^†^	61.80(52.90-80.20)^†^	0.758	60.20(52.95-80.90)^†^	62.20(51.60-75.10)^†^	0.913
Non-HDL-C, mg/dL	75.00(69.50-99.00)^†^	78.50(65.75-92.50)^†^	0.546	74.50(69.75-98.00)^†^	79.00(66.50-93.00)^†^	0.799
HDL-C, mg/dL	36.00(34.00-41.00)^†^	40.00(35.00-46.00)^†^	**0.004**	36.00(34.00-41.00)^†^	40.0035.00-45.00)^†^	**0.011**
TG, mg/dL	84.00(60.00-106.50)^†^	71.50(56.00-89.25)^†^	0.051	82.50(59.00-106.25)^†^	76.00(57.00-91.50)^†^	0.252
TG/HDL-C	2.13(1.90-2.64)^†^	1.93(1.47-2.36)^†^	**0.005**	2.11(1.89-2.73)^†^	2.00(1.49-2.41)^†^	**0.024**

SNPs: Single nucleotide polymorphism; *CETP*: cholesteryl ester transfer protein; SCD: sickle cell disease; n: number of participants; ApoA1: apolipoprotein A1; ApoB: apolipoprotein B; TC: total cholesterol; LDL-C: low-density lipoprotein cholesterol; Non-HDL-C: non-high-density lipoprotein cholesterol; HDL-C: high-density lipoprotein cholesterol; TG: triglycerides; TG/HDL-C: triglyceride/high-density lipoprotein cholesterol ratio. *Results of the Student's *t*-test are reported as means±SD; ^†^Results of the Mann Whitney's U-test are reported as median (first and third quartiles: Q1-Q3). Significant results (P<0.05) are shown in bold.

**Figure 1:**
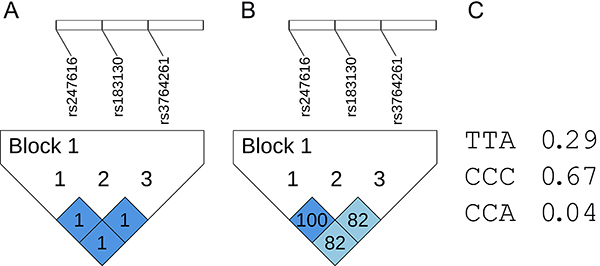
Graphical representation of the haplotype with high linkage disequilibrium and r^2^ of the single nucleotide polymorphism haplotypes of the *CETP* gene. **A**, Linkage disequilibrium, D'=1; **B**, r^2^. (1) rs247616 (C>T), (2) rs183130 (C>T), and (3) rs3764261 (C>A). **C**, Identified haplotypes and associated frequencies. Analyses were performed with the Haploview software (https://www.broadinstitute.org).

The analyses in the Haploview software (Figure 1) showed a high linkage disequilibrium between the three variants of the *CETP* gene (rs183130 and rs247616: D'=1.0 and R^2^=1.0; rs183130 and rs3764261: D'=1.0 and R^2^=0.82; rs247616 and rs3764261: D'=1.0 and R^2^=0.82). A D' equal to 1 signifies that the alleles of the SNPs are inherited together more frequently than expected by chance alone and a higher r^2^ value suggests a stronger correlation between the SNPs, indicating that they are inherited together more consistently. It was possible to identify in the SCD population a haploblock formed from the three *CETP* variants (CCC, TTA, and CCA). The frequency of haplotypes CCC, TTA, and CCA was 0.67, 0.29, and 0.04, respectively. The presence of the TTA haplotype was associated with high HDL-C (≥40 mg/dL; P=0.015) and low TG/HDL-C ratio (≤2.0; P=0.047).

### Explanatory variables of HDL-C levels

Considering the previous results related to HDL-C and to identify which markers would explain the alteration of this lipoprotein, a binary logistic regression analysis was performed, including HDL-C (case <40 mg/dL and control ≥40 mg/dL) as a dependent variable and a *CETP* variant and a laboratory marker of severity as an independent variable in each analysis. Age was included in the analysis to adjust the models. The absence of minor alleles of the three variants of the *CETP* gene (allele A of rs3764261 and T of rs183130 and rs247616) in the genotype dominance model increased the odds for low HDL-C of the individuals with SCD by approximately three times (Table 6).

**Table 6:** Association of hemoglobin level and *CETP* variants with the HDL-cholesterol levels in participants with sickle cell disease (n=131).

Variables	OR (95%CI)	P value
Model 1		
Hemoglobin level	0.79 (0.64-0.97)	**0.026**
CC genotype of rs247616 and rs183130*	3.60 (1.60-8.08)	**0.002**
Age	1.17 (1.01-1.36)	**0.032**
Model 2		
Hemoglobin level	0.75 (0.61- 0.94)	**0.012**
Genotype CC of rs3764261*	3.04 (0.14-6.82)	**0.007**
Age	1.17 (1.01-1.35)	**0.036**

*CETP*: cholesteryl ester transfer protein; HDL-cholesterol: high-density lipoprotein cholesterol; SCD: sickle cell disease. *The reference group for categorical variable was: genotypes TT+CT for rs247616 and rs183130 and genotypes AA+CA for rs3764261; case: HDL-C <40 mg/dL and control: HDL-C ≥40 mg/dL. OR: odds ratio; CI: confidence interval. Binary logistic regression analysis: significant results (P<0.05) are shown in bold.

The effect of the TTA haplotype on HDL-C levels was evaluated considering two groups of genotypes, which were organized according to the presence or absence of the TTA haplotype (genotypes CCC/CCC, CCC/ACC *vs* TTA/TTA, CCC/TTA, and ACC/TTA). The odds of having low HDL-C levels was 3.4 times higher in the absence of the TTA haplotype in the genotype, and this effect was maintained after adjustment by HU therapy (P=0.002) (Table 7).

**Table 7:** Association of hemoglobin level and absence of the TTA haplotype of the *CETP* gene with levels of HDL-cholesterol in participants with sickle cell disease (n=131).

Variables	OR (95%CI)	P value
Model 1		
Hemoglobin level	0.74 (0.60- 0.93)	**0.009**
*Genotypes CCC/CCC, CCC/ACC	3.43 (1.53-7.71)	**0.003**
Age	1.17 (1.01-1.36)	**0.031**
Model 2		
Genotypes CCC/CCC, CCC/ACC	3.50 (1.55-7.90)	**0.002**
Age	1.17 (1.01-1.36)	**0.040**
Hydroxyurea	1.43 (0.60-3.37)	0.411

*CETP*: cholesteryl ester transfer protein; HDL-cholesterol: high-density lipoprotein cholesterol; SCD: sickle cell disease. Analyses performed with haplotype genotypes grouped by presence or absence of TTA and hemoglobin as independent variables. *Absence of haplotype TTA; Reference: presence of TTA haplotype in genotype. Case: HDL-C <40 m/dL and control: HDLC ≥40 mg/dL. Hydroxyurea (yes or no) was used to adjust the model. OR: odds ratio; CI: confidence interval. Logistic regression analysis: models; significant results (P<0.05) are shown in bold.

In turn, higher Hb levels were associated with higher HDL-C levels (Tables 6 and 7). HU therapy was not significant in the model (Table 7). Other laboratory markers of severity evaluated were also not significant.

## Discussion

In this study, we investigated the relationship between lipid fractions and demographic and anthropometric variables, alpha-thalassemia, HU therapy status, and laboratory markers of severity. The interaction with polymorphisms and haplotypes in the *CETP* gene in a pediatric population with SCD were also investigated.

Overall, we found higher TG and cholesterol levels in the SCA group compared to the SCC. However, the SCD genotype did not influence HDL-C and ApoA1 levels. The differences in lipid profile between SCA and SCC have been studied in a limited number of papers (6,22-25), and a lack of agreement in the results was observed. The divergence regarding age group may justify the differences. Lower levels of total cholesterol and HDL-C have been found in the SCA group than in the SCC group in studies where there is important inclusion of adults (6,24). However, the studies by Santiago et al. (23) and Ephraim et al. (25), which included at least seventy percent of individuals under 20 years of age, show no significant difference in total cholesterol levels between SCA and SCC. In the case of HDL-C, studies consistently indicate lower levels in the SCA group (6,22-25), although it did not reach significance in the study by Ephraim et al. (25).

Despite the association between BMI and HDL-C in the B razilian pediatric population without SCD (26), this relationship was not observed in the present study, which is in line with the results observed by Ephraim et al. (25). The lower BMI z-score values and the lower frequency of overweight make it difficult to infer this relationship.

Dyslipidemia has been considered one of the subphenotypes of SCD, with hypocholesterolemia being a common characteristic in this population, differently than individuals without SCD (7,23,27,28). The mechanism for this change is not clear, but inflammation, oxidative stress, and chronic hemolysis have been indicated as involved factors (4,23). In our population with SCD, higher bilirubin and white blood cell values and a positive association of HDL-C levels with Hb were observed in the group with lower ApoA1 and HDL-C levels. These results reinforce the relationship between hypocholesterolemia and SCD severity observed in previous studies (22,29).

A higher TG/HDL-C ratio (>2) was associated with higher values of bilirubin, white blood cells, platelets, and LDH and lower Hb values. In children and adolescents with SCA, the TG/HDL-C ratio was considered a potential marker of vascular events, since it was positively associated with white blood cells, endothelial dysfunction, and higher blood flow velocity in the cerebral arteries (3).

Our study revealed that individuals with SCD and coexisting alpha-thalassemia exhibited lower TG levels. Alpha-thalassemia is a hemoglobinopathy characterized by a deficiency in the production of α-globin chains, and it appears to have an impact on the lipid profile of individuals with SCD (30), although different effects have been reported. Valente-Frossard et al. (29) observed lower total cholesterol levels in children and adolescents with SCD who also had alpha-thalassemia. However, Aleluia et al. (6) found no differences in the lipid profile between individuals with and without α^3.7^-thalassemia in SCA. It is well-established that markers of intravascular hemolysis, vascular dysfunction, and pulmonary hypertension in SCD patients are associated with dysregulated plasma lipids, including elevated TG levels (31). In turn, inflammatory and oxidative processes are associated with alterations in metabolism and lipid peroxidation products in SCD (32). While SCD is primarily marked by vaso-occlusive crises, hemolysis, and organ damage, the presence of alpha-thalassemia has been linked to phenotypic modifications, potentially serving as a modifier factor for some of these manifestations (33). Studies have shown that alpha-thalassemia has a modulatory effect on oxidative stress in SCA, likely attributed to a decrease in myeloperoxidase (MPO) activity (34). MPO is an enzyme involved in oxidative and inflammatory processes, and it has been demonstrated to cause oxidative damage to ApoA1 (4). Triglyceride-rich lipoproteins (apoB-containing lipoproteins) have been shown to increase inflammatory markers and MPO within the arterial wall (35). Such a modifying effect of alpha-thalassemia in MPO may provide a possible explanation for our findings of alpha-thalassemia and low levels of TG in SCD. Another potential explanation is the impact of altered erythropoiesis on lipid metabolism. Alpha-thalassemia affects red blood cell production and can lead to ineffective erythropoiesis, where a higher number of immature red blood cells are produced (36). This altered erythropoiesis may disrupt normal lipid metabolism and result in lower levels of TG as observed in the coexistence of alpha-thalassemia in SCD. It should be emphasized that these explanations are hypothetical and more research is needed to fully understand the relationship between alpha-thalassemia and low levels of TG. Additionally, individual genetic variations, environmental factors, and other coexisting conditions may influence this association.

Studies of the effects of HU therapy on lipid profiles are scarce. HU improves hemolytic and inflammatory parameters, reducing disease severity and thus it is commonly used in more severe individuals, especially in SCA (37). This may explain the higher levels of ApoA1 and HDL-C, as well as the similarity in TG levels observed in individuals using HU in our research. Teixeira et al. (5) found no effects of HU therapy on HDL-C levels when investigating children and adolescents with SCA.

Previous studies have demonstrated a correlation between elevated HDL-C levels and *CETP* gene variations in individuals with and without chronic diseases (11,13,14,16). In our investigation, we examined the interaction of specific *CETP* variants (rs247616, rs183130, and rs3764261) with lipid profile in SCD. Our findings indicated that these SNPs independently contribute to HDL-C levels, both in the dominance model of biallelic genotypes and when evaluating haplotypes. Interestingly, the absence of TTA haplotype was associated with a three-fold increase in the odds of low HDL-C levels. CETP activity is reduced (11,15,38) and HDL-C levels are increased (11,12) in the presence of the minor allele frequencies of the analyzed SNPs. In adults without SCD, an increase of 0.32 μg/mL was observed in serum CETP levels for the rs247616-C SNP (11). McCaffery et al. (13) observed that each copy of the rs3764216 risk allele C was associated with lower baseline HDL-C. Additionally, the rare alleles of rs3764216 and rs183130 were associated with low HDL-C levels in American white and African black populations (14). Based on these findings, we hypothesized that the TTA haplotype likely reduced the CETP level or expression, leading to increased HDL-C levels and a lower TG/HDL-C ratio in individuals with SCD.

Although low HDL-C levels may be a consequence of severe hemolytic anemia, the absence of minor alleles of the *CETP* gene in patients with SCD also proved to be an explanatory pathway for the observed hypocholesterolemia. In this way, this genetic marker may function as a modulating factor of HDL-C levels together with the pathophysiological effects of anemia itself. This shows that the dyslipidemia observed in SCD in several populations can be minimized or worsened depending on the co-inheritance of genetic variants involved in lipid metabolism or, conversely, be a confounding factor in the interpretations about the pathophysiological mechanism of dyslipidemia in anemia. Therefore, the *CETP* gene variants added to the intrinsic factors of hemolytic anemia itself (cumulative effect of chronic hemolysis, oxidative stress, and inflammation) could explain part of the variation observed in dyslipidemia in patients with SCD.

To our knowledge, this is the first study involving analysis of haplotypes with the three variants in the *CETP* gene in individuals with SCD, and no reports of these haplotypes have been found in other diseases. This study extends the knowledge about dyslipidemia and pathophysiological aspects resulting from lipid homeostasis in SCD. However, current explanations for this particular sub-phenotype are neither comprehensive nor consensual.

The study had limitations such as the absence of information about clinical manifestations, measurements of plasma levels of CETP, and oxidative parameters. However, the observed results are sufficient to achieve the objectives proposed in the study.

Our findings suggested that the dyslipidemia commonly observed in SCD, particularly in relation to HDL-C levels, may not be due solely to the disease itself but rather to a genetic background carried by the individual. Additional and prospective studies with a representative population are necessary to confirm the associations observed in this paper and identify the future impacts of dyslipidemia on the clinical severity of SCD and cardiovascular repercussions in adulthood. Furthermore, it is important to determine whether clinical manifestations are influenced by the presence of *CETP* gene variants. This broader understanding can help unravel the complex interplay between genetic factors, lipid profiles, and clinical outcomes in SCD.

## Supplementary Material

Click to view [pdf].
