# Supplementary material for: CETP gene polymorphisms and haplotypes are explanatory variables for HDL cholesterol level in sickle cell disease
Source: Braz J Med Biol Res. 2024 Jan 22;57:e12879. doi: 10.1590/1414-431X2023e12879 (PMC10802225; doi:10.1590/1414-431X2023e12879)
Supplement: Supplementary file 1 [file 1414-431X-bjmbr-57-e12879-suppl.pdf]

Table S1. Correlation of lipid profile with BMI z-score and laboratory markers of severity in participants with sickle cell disease.

Variables	BMI-Z n=130	Hb n=122	Platelets n=118	WBC n=118	TB n=127	DB n=127	IB n=127	LDH n=118
ApoA1								
r	0.157	0.219	–0.038	–0.246	–0.244	–0.127	–0.247	–0.204
P	0.073	**0.015**	0.683	**0.007**	**0.005**	0.150	**0.005**	**0.020**
ApoB								
r	0.032	–0.108	0.220	0.027	0.016	0.108	0.001	0.125
P	0.718	0.231	**0.016**	0.767	0.859	0.223	0.993	0.157
TC								
r	0.075	0.066	0.206	–0.053	–0.058	0.051	–0.071	0.089
P	0.391	0.467	**0.024**	0.562	0.514	0.566	0.427	0.317
LDL-C								
r	0.051	–0.025	0.160	–0.079	–0.084	0.014	–0.095	0.034
P	0.564	0.785	0.082	0.393	0.342	0.871	0.286	0.704
Non-HDL-C								
r	0.075	–0.146	0.223	0.037	0.019	0.098	0.005	0.145
P	0.391	0.106	**0.014**	0.688	0.831	0.267	0.953	0.100
HDL-C								
r	0.059	0.231	–0.045	–0.271	–0.206	–0.111	–0.208	–0.152
P	0.501	**0.010**	0.622	**0.003**	**0.019**	0.209	**0.018**	0.086
TG								
r	–0.036	–0.353	0.219	0.316	0.265	0.225	0.258	0.322
p	0.683	**<0.001**	**0.016**	**<0.001**	**0.002**	**0.011**	**0.003**	**<0.001**
TG/HDL-C								
r	–0.067	–0.249	0.267	0.222	0.133	0.138	0.125	0.224
P	0.443	**0.005**	**0.003**	**0.015**	0.133	0.119	0.159	**0.011**

BMI-Z: body mass index z-score; Hb: hemoglobin; WBC: white blood cells; TB: total bilirubin; DB: direct bilirubin; IB: indirect bilirubin; LDH: lactate dehydrogenase; n: number of participants; ApoA1: apolipoprotein A1; ApoB: apolipoprotein B; TC: total cholesterol; LDL-C: low-density lipoprotein cholesterol; Non-HDL-C: non-high-density lipoprotein cholesterol; HDL-C: high-density lipoprotein cholesterol; TG: triglycerides; TG/HDL-C: triglycerides/high-density lipoprotein cholesterol ratio. Results of the Spearman/Partial correlation test. Partial analysis controlling for the effect of age. Statistically significant results ($P<0.05$) are shown in bold.

Table S2. Severity markers and the dichotomized values of the lipid profile in participants with sickle cell disease.

Severity markers	n	ApoA1 <115 mg/dL	n	ApoA1 ≥115 mg/dL	P	n	HDL-C <40 mg/dL	n	HDL-C ≥40 mg/dL	P
Hemoglobin, g/dL	89	9.30 (7.85–11.20)†	36	11.15 (9.70–12.02)†	**<0.001**	75	9.49 (2.02)*	50	10.28 (1.79)*	**0.023**
WBC, $10^3/mm^3$	88	9.08 (4.20)*	33	7.44 (3.02)*	**0.042**	74	9.30 (4.27)*	47	7.59 (3.22)*	**0.021**
Platelets, $\times 10^3/mm^3$	88	411.50 (210.50–508.25)†	33	329.00 (216.50–488.50)†	0.394	74	406.50 (213.50–521.25)†	47	370.00 (199.00–488.00)†	0.436
Total bilirubin, mg/dL	90	1.88 (1.14–2.84)†	40	1.21 (0.98–2.05)†	**0.019**	76	1.88 (1.11–2.84)†	54	1.33 (1.02–2.37)†	0.107
Direct bilirubin, mg/dL	90	0.48 (0.18)*	40	0.41 (0.14)*	**0.010**	76	0.48 (0.18)*	54	0.44 (0.15)*	0.200
Indirect bilirubin, mg/dL	90	1.38 (0.71–2.25)†	40	0.84 (0.66–1.47)†	**0.044**	76	1.38 (0.73–2.31)†	54	0.90 (0.66–1.75)†	0.104
LDH U/L	90	432.50 (279.00–613.75)†	40	308.50 (258.25–425.00)†	**0.011**	76	399.00 (276.00–561.75)†	54	334.00 (264.00–553.50)†	0.325
	n	TG <100/130 mg/dL	n	TG ≥100/130 mg/dL		n	TG/HDL-C ≤2	n	TG/HDL-C >2	
Hemoglobin, g/dL	109	10.00 (1.90)*	16	8.46 (1.97)*	**0.008**	58	10.36 (1.92)*	67	9.33 (1.90)*	**0.003**
WBC, $10^3/mm^3$	105	7.90 (6.10–11.1)†	16	11.55 (5.92–13.75)†	0.070	54	7.55 (3.41)*	67	9.51 (4.20)*	**0.006**
Platelets, $\times 10^3/mm^3$	105	364.00 (205.50–484.00)†	16	504.50 (320.50–609.75)†	**0.035**	54	320.50 (181.50–479.00)†	67	430.00 (259.00–525.00)†	**0.029**
Total bilirubin, mg/dL	112	1.62 (1.07–2.61)†	18	2.08 (0.96–3.61)†	0.377	58	1.21 (1.00–2.33)†	72	1.98 (1.20–2.95)†	**0.007**
Direct bilirubin, mg/dL	112	0.45 (0.16)*	18	0.52 (0.21)*	0.187	58	0.41 (0.16)*	72	0.50 (0.17)*	**0.006**
Indirect bilirubin, mg/dL	112	1.25 (0.71–2.02)†	18	1.56 (0.60–3.10)†	0.597	58	0.80 (0.65–1.76)†	72	1.45 (0.76–2.33)†	**0.017**
LDH, U/L	112	382.50 (362.00–519.00)†	18	427.00 (304.50–803.25)†	0.133	58	314.00 (256.00–430.25)†	72	448.00 (316.75–628.25)†	**0.002**

Data are reported as means±SD or median (first and third quartiles, Q1–Q3). n: number of participants; ApoA1: apolipoprotein A1; HDL-C: high-density lipoprotein cholesterol; TG: triglycerides; TG/HDL-C: triglyceride/high-density lipoprotein cholesterol ratio; WBC: white blood cells; LDH: lactate dehydrogenase. Levels of triglycerides below and above 10 years of age (≥100 or 130 mg/dL, respectively). Statistically significant results (P<0.05) are shown in bold: *Student's *t*-test, †Mann Whitney's U-test.
